# Formation of tRNA Wobble Inosine in Humans Is Disrupted by a Millennia-Old Mutation Causing Intellectual Disability

**DOI:** 10.1128/MCB.00203-19

**Published:** 2019-09-11

**Authors:** Jillian Ramos, Lu Han, Yan Li, Felix Hagelskamp, Stefanie M. Kellner, Fowzan S. Alkuraya, Eric M. Phizicky, Dragony Fu

**Affiliations:** a Department of Biology, University of Rochester, Rochester, New York, USA; b Center for RNA Biology, University of Rochester and University of Rochester Medical Center, Rochester, New York, USA; c Department of Biochemistry and Biophysics, University of Rochester Medical Center, Rochester, New York, USA; d Department of Chemistry, Ludwig Maximilians Universität München, Munich, Germany; e Department of Genetics, King Faisal Specialist Hospital and Research Center, Riyadh, Saudi Arabia; f Department of Anatomy and Cell Biology, College of Medicine, Alfaisal University, Riyadh, Saudi Arabia

**Keywords:** RNA editing, adenosine deaminase, inosine, intellectual disability, molecular genetics, neurodevelopment, tRNA, tRNA modification

## Abstract

The formation of inosine at the wobble position of eukaryotic tRNAs is an essential modification catalyzed by the ADAT2/ADAT3 complex. In humans, a valine-to-methionine mutation (V144M) in ADAT3 that originated ∼1,600 years ago is the most common cause of autosomal recessive intellectual disability (ID) in Arabia. While the mutation is predicted to affect protein structure, the molecular and cellular effects of the V144M mutation are unknown. Here, we show that cell lines derived from ID-affected individuals expressing only ADAT3-V144M exhibit decreased wobble inosine in certain tRNAs. Moreover, extracts from the same cell lines of ID-affected individuals display a severe reduction in tRNA deaminase activity. While ADAT3-V144M maintains interactions with ADAT2, the purified ADAT2/3-V144M complexes exhibit defects in activity. Notably, ADAT3-V144M exhibits an increased propensity to form aggregates associated with cytoplasmic chaperonins that can be suppressed by ADAT2 overexpression. These results identify a key role for ADAT2-dependent folding of ADAT3 in wobble inosine modification and indicate that proper formation of an active ADAT2/3 complex is crucial for proper neurodevelopment.

## Abstract (precis)

The formation of inosine at the wobble position of eukaryotic tRNAs is an essential modification catalyzed by the ADAT2/ADAT3 complex. In humans, a valine-to-methionine mutation (V144M) in ADAT3 that originated ∼1,600 years ago is the most common cause of autosomal recessive intellectual disability (ID) in Arabia. While the mutation is predicted to affect protein structure, the molecular and cellular effects of the V144M mutation are unknown.

## INTRODUCTION

The hydrolytic deamination of adenosine (A) to inosine (I) at the wobble position of tRNA is an essential posttranscriptional tRNA modification in bacteria and eukaryotes (1, 2). Since inosine can pair with U, C, or A, a single tRNA isoacceptor containing the inosine modification at the wobble anticodon position can recognize up to three different codons containing a different nucleotide base at the third position. Thus, the degeneracy provided by the wobble inosine modification is necessary for the translation of C- or A-ending codons in organisms that lack a cognate G_34_- or U_34_-containing anticodon tRNA isoacceptor by expanding the reading capacity of tRNA isoacceptors (3). Moreover, it has been shown that highly translated genes in eukaryotic organisms, including humans, are correlated with an enrichment in wobble inosine tRNA-dependent codons, suggesting a critical role for tRNA inosine modification in maintaining proper levels of protein expression (4, 5).

In Escherichia coli, A-to-I conversion at the wobble position is present in a single tRNA (tRNA-Arg-ACG) and is catalyzed by the homodimeric complex TadA adenosine deaminase (1). In the yeast Saccharomyces cerevisiae, wobble inosine modification occurs in seven different tRNAs and is catalyzed by a heterodimeric enzyme complex consisting of the Tad2p and Tad3p subunits (2, 6). Tad2p is the catalytic subunit and contains a prototypical deaminase motif homologous to cytidine/deoxycytidine deaminases, including a conserved glutamic acid residue within the active site that is necessary for proton shuttling in the hydrolytic deamination reaction (7, 8). Tad3p also contains a canonical deaminase motif but lacks the conserved catalytic glutamate in the active site. However, Tad2p is inactive without Tad3p, indicating that formation of a heterodimeric Tad2p/Tad3p complex is required for adenosine deaminase activity (2). Functional homologs of S. cerevisiae Tad2p and Tad3p have been identified in all eukaryotes to date, including the human homologs ADAT2 and ADAT3 (9–12).

Exome sequencing and autozygosity mapping have identified a single c.382G>A mutation in the human *ADAT3* gene that is causative for autosomal recessive intellectual disability (ID) in multiple families of Saudi Arabian descent (13–15). All reported individuals homozygous for the V144M mutation exhibit cognitive deficits indicative of a neurodevelopmental disorder, with the majority displaying strabismus and growth delay. Additional clinical features of individuals homozygous for the ADAT3-V144M mutation include microcephaly, epilepsy, and occasional brain abnormalities such as white matter atrophy and arachnoid cysts. Subsequent large-scale sequencing has identified this ancient founder mutation to be one of the most common causes of autosomal recessive intellectual disability in patients from Saudi Arabia, with a carrier frequency of ∼1% (16–18). However, the mechanistic cause of ADAT3-associated pathogenesis remains unclear.

The human *ADAT3* gene expresses two mRNA transcripts encoding ADAT3 proteins that differ only by the addition of 16 amino acid residues to the amino terminus of the longer ADAT3 isoform. Based upon the longer ADAT3 isoform, the ID-causing G>A transition results in a valine-to-methionine missense mutation at residue 144 (V144M). The mutated valine residue is conserved from yeast to humans and is predicted to perturb the surface structure of the ADAT3 protein (13). However, it is unknown how the V144M mutation affects ADAT3 function and whether this would affect tRNA inosine modification levels in ID-affected individuals who are homozygous for the autosomal recessive mutation. This would be important to test given the increasing awareness of tRNA modification in the etiology of other forms of Mendelian ID (19–30).

Here, we demonstrate that cells isolated from ID-affected individuals homozygous for the ADAT3-V144M mutation contain diminished levels of wobble inosine in several tRNA isoacceptors. Moreover, we find that extracts from these cells exhibit a drastic decrease in adenosine deaminase activity. While the ADAT3-V144M variant can form complexes with its heterodimeric partner ADAT2, ADAT2/3-V144M complexes exhibit greatly reduced enzymatic activity and an increased propensity to self-associate. Using subcellular localization studies combined with proteomics, we find that overexpressed ADAT3-V144M exhibits aberrant aggregation into cytoplasmic foci accompanied by targeting by the heat shock protein 60 (HSP60) and TRiC/CCT chaperonin complexes. Notably, the aggregation phenotype of ADAT3-V144M along with its association with chaperonins can be suppressed by coexpression with ADAT2. Altogether, these results uncover a potential molecular basis for ADAT3-associated neurodevelopmental disorders in the form of diminished inosine modifications at the wobble position of tRNA caused by ADAT3 misfolding and impaired enzymatic activity.

## RESULTS

### Individuals homozygous for the ADAT3-V144M mutation exhibit decreased wobble inosine modification in tRNAs.

**FIG 1:**
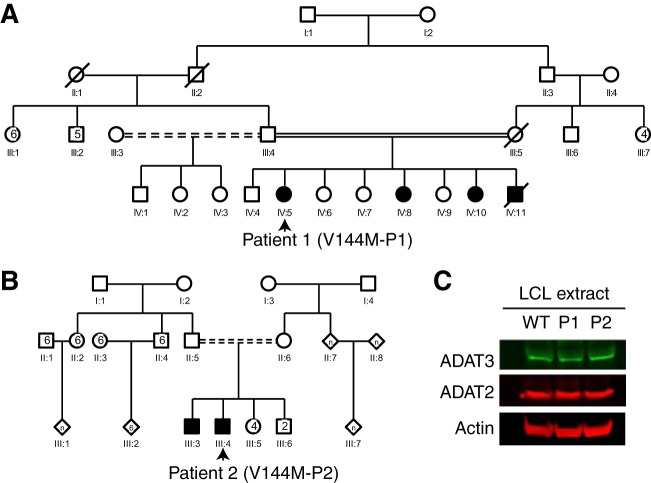
Individuals homozygous for the ADAT3-V144M mutation exhibit similar levels of ADAT3 expression. (A and B) Pedigrees of patient 1 (P1) and P2 containing homozygous V144M missense mutations in the ADAT3 gene. Male family members are denoted by squares, females are denoted by circles, individuals of unknown sex are denoted by diamonds, deceased individuals are denoted by slashes, ID-affected individuals with homozygous V144M mutations are denoted by shading, and consanguinity is denoted by double solid or shaded lines. (C) Immunoblot for the indicated proteins of extracts from LCLs donated from a wild-type (WT) individual and P1 and P2 harboring homozygous V144M mutations.

To examine the molecular effects of the V144M mutation in the human population, we generated lymphoblastoid cell lines (LCLs) from two unrelated human patients harboring homozygous V144M missense mutations in the *ADAT3* gene (referred to as V144M-LCLs, generated from patient 1 [P1] and P2) (Fig. 1A and B). P1 is a 6-year-old female with severe ID, short stature, microcephaly, strabismus, deafness, and a history of global developmental delay (13, 16). She is part of a consanguineous family with three similarly affected siblings (Fig. 1A). P2 is a 24-year-old male with features similar to those of P1, including severe ID, microcephaly, and developmental deficits as a child. P2 is also part of a consanguineous family with a similarly affected brother (Fig. 1B) (14). LCLs generated from both ID-affected individuals with homozygous V144M mutations were compared to control lymphoblasts from an ethnically matched, healthy, unrelated individual (WT1-LCLs).

We first examined the levels of ADAT3 protein in LCLs to determine if the expression or stability of ADAT3 was affected by the V144M mutation. Based upon immunoblotting of whole-cell lysates, no major change in the endogenous levels of ADAT3 was detected between wild-type LCLs (WT-LCLs) and V144M-LCLs (Fig. 1C). Moreover, the levels of the ADAT3 heterodimeric binding subunit, ADAT2, were also similar between WT- and V144M-LCLs (Fig. 1C). The comparable steady-state levels of wild-type ADAT3 and the V144M variant suggest that the V144M mutation could be impacting ADAT3 function without affecting protein accumulation.

**FIG 2:**
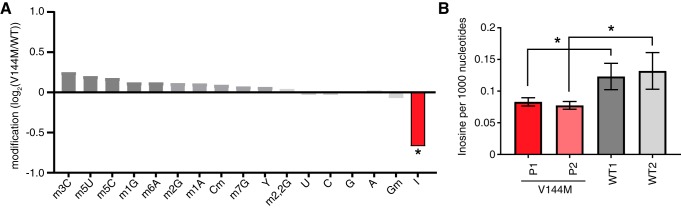
ID-affected individuals expressing only the ADAT3-V144M variant exhibit decreased wobble inosine modification in tRNA isoacceptors. (A) Comparison of tRNA modification levels between V144M- and WT-LCLs. Nucleosides from digested tRNA samples were analyzed by LC-MS. The *y* axis represents the log_2_ fold change in the levels of the indicated tRNA modifications between the two patients harboring homozygous V144M mutations described in the legend of Fig. 1A and two WT individuals. (B) Inosine modification levels in total tRNA from LCLs of two WT individuals and P1 and P2 harboring homozygous V144M mutations. Inosine levels were measured by absolute quantification to calculate the number of inosines per 1,000 nucleotides. The means and error bars represent measurements from 3 independent RNA samples from each cell line. *, *P* < 0.05.

We next monitored the levels of tRNA modifications in WT- versus V144M-LCLs using liquid chromatography-mass spectrometry (LC-MS) of nucleosides from digested total tRNA (31). For these analyses, we also performed a comparison against a completely different LCL procured from a healthy individual of a similar age but a different ethnic background (WT2-LCLs). Among 13 tRNA modifications tested, we found that the inosine modification differed the greatest between WT- and V144M-LCLs, with V144M LCLs exhibiting a substantial decrease in tRNA inosine levels (Fig. 2A). Using absolute quantification by LC-MS with calibration standards, we found that the abundance of inosine was reduced by ∼30% in the tRNAs of both V144M-LCLs compared to either WT-LCL (Fig. 2B). While we detected a significant decrease in the levels of inosine in the tRNA of V144M-LCLs (*P* < 0.05), no significant change was detected in the levels of any other tested modification (*P* > 0.1). These results provide the first evidence that the ADAT3-V144M mutation and its associated molecular defects have an impact on the levels of tRNA wobble inosine modification *in vivo*.

**FIG 3:**
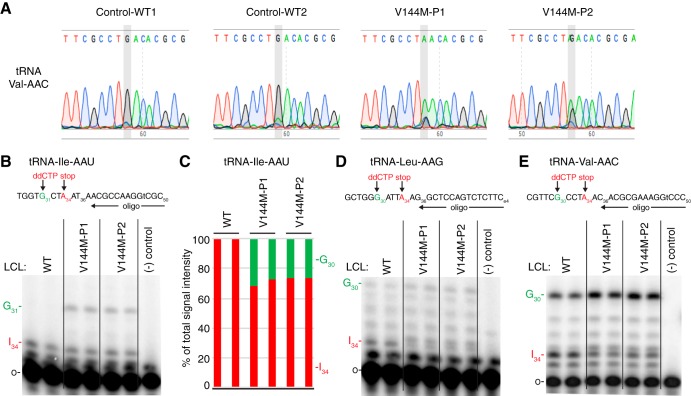
ID-affected individuals expressing only the ADAT3-V144M mutant exhibit a reduction in wobble inosine modification in tRNA isoacceptors. (A) Sequencing chromatogram analysis of RT-PCR products amplified from endogenous tRNA-Val-AAC isolated from LCLs of the indicated individuals. The wobble adenosine/inosine position is highlighted in gray. Inosine is read out as G. (B, D, and E) V144M-LCLs exhibit decreased inosine modification in tRNA-Ile-AAU, -Val-AAC, and -Leu-AAG. Primer extension analysis was performed with the indicated oligonucleotide probes against inosine-containing tRNAs in the presence of ddCTP. “G*_n_*” denotes a readthrough product indicative of decreased inosine modification at position 34. “I_34_” represents the stop position if inosine is present. “o” represents the labeled oligonucleotide used for primer extension. (−) control represents a primer extension reaction without the addition of reverse transcriptase. (C) Quantification of primer extension for tRNA-Ile-AAU. The RT stop signal due to inosine modification and the G_31_ readthrough product are plotted as a fraction of the total signal intensity for both bands.

Focusing on a specific tRNA, we next investigated the wobble inosine status of tRNA-Val-AAC isolated from the V144M-LCLs of ID-affected individuals. Since inosine is read as G by reverse transcriptases (32–34), the formation of inosine at the wobble position of tRNA-Val-AAC can be directly detected by sequencing of amplified cDNA obtained by reverse transcription (RT) of cellular tRNA. In WT-LCLs, the majority of wobble adenosines in tRNA-Val-AAC were converted to inosine, as evidenced by the presence of a predominant “G” peak at position 34 (Fig. 3A). Notably, the level of wobble inosine modification in tRNA-Val-AAC was greatly reduced in both V144M-LCLs, with the majority of the peak signal at the wobble position being the unmodified “A” (Fig. 3A).

Based upon the inosine modification defect in tRNA-Val-AAC, we also investigated whether additional tRNAs containing inosine at the wobble position were affected by the ADAT3-V144M mutation. Due to technical challenges in RT-PCR sequencing analysis caused by the diverse number of tRNA isodecoder variants encoded by mammalian genomes, we investigated the modification status of human tRNAs using poisoned primer extension assays with a ddCTP terminator, to distinguish I_34_ (terminated with ddCTP) from A_34_, terminated at the next guanosine (Fig. 3B to E). Using this assay, we observed a reduced frequency of I_34_ modification in tRNA-Ile-AAU, from nearly 100% to ∼70% for P1 and 75% for P2 (Fig. 3B and C). For tRNA-Val-AAC and tRNA-Leu-AAG, we also observed a reduction in I_34_ in the tRNAs from both patients (Fig. 3D and E). Although accurate quantification of deamination for tRNA-Val-AAC and tRNA-Leu-AAG was not possible because of the high background signal in the primer extensions, the reduced I_34_ signal and consequent increase in readthrough products up to G_30_ were indicative of reduced inosine modification. These studies uncover a wobble inosine hypomodification defect for particular tRNAs in the cells of individuals who are homozygous for the ADAT3-V144M mutation. Moreover, while the ADAT3-V144M mutation reduces the deamination of multiple tRNAs, the effect is incomplete and not necessarily to the same extent in all affected tRNAs.

### Human patient cells with homozygous ADAT3-V144M mutations exhibit perturbations in cellular tRNA adenosine deaminase activity.

We next investigated whether the V144M mutation affects adenosine deaminase activity in the cells of ID-affected individuals expressing only the ADAT3-V144M variant. We performed an *in vitro* adenosine deaminase activity assay using whole-cell extracts prepared from the human LCLs described above. This was made possible since previous studies have shown that the activity of the ADAT2/3 enzyme complex is the only known cellular activity that catalyzes wobble inosine formation in tRNA (6). To detect inosine formation, we used an adenosine deaminase assay based upon the separation of digested RNA nucleoside products by thin-layer chromatography (TLC) (35, 36). For this assay, *in vitro*-transcribed tRNA substrates were internally radiolabeled at adenosine residues using [α-^32^P]ATP and incubated with the whole-cell extract from either the wild-type or V144M individuals, and the RNAs were digested to nucleoside monophosphates with P1 nuclease followed by TLC separation to detect IMP formation. As the substrate, we used human tRNA-Val-AAC, since it has been shown to be a target of ADAT2/3-catalyzed deamination *in vitro* and *in vivo* (12).

**FIG 4:**
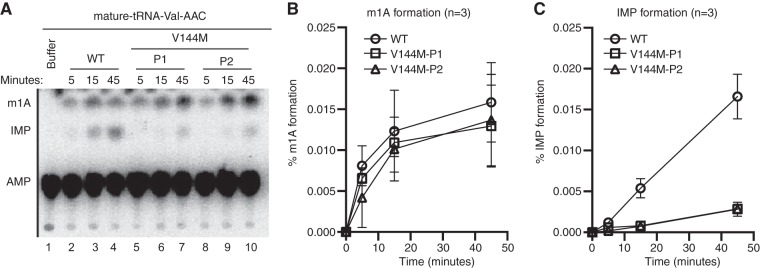
Individuals homozygous for the ADAT3-V144M mutation exhibit defects in adenosine deaminase activity. (A) Representative time course assay of adenosine deaminase activity using human cellular extracts and mature tRNA-Val-AAC from 5 to 45 min. (B and C) Quantification of m1A or IMP formation as a function of time for the indicated cellular extracts prepared from human LCLs. Percent m1A or IMP formation represents either the m1A/AMP+m1A+IMP or the IMP/AMP+m1A+IMP signal (*n* = 3).

While no detectable IMP was detected in tRNA preincubated with buffer alone (Fig. 4A, lane 1), we could readily detect the formation of IMP in tRNA-Val-AAC after preincubation with whole-cell extracts prepared from WT-LCLs (Fig. 4A, lanes 2 to 4). In addition to IMP, we also detected a faster-migrating adenosine modification that is consistent with the formation of 1-methyladenosine (m1A) (Fig. 4A) (37). Since human tRNA-Val-AAC has m1A at position 58 (38), the formation of m1A is likely due to endogenous TRMT6/TRMT61 complexes present in cellular extracts (39). The formation of m1A provides an internal control for cellular adenosine deaminase activity since it is catalyzed by two different enzyme complexes. Using a time course to monitor product formation, we detected similar levels of m1A formation between WT- and V144M-LCL extracts (Fig. 4B). In contrast, we found that V144M-LCL extracts exhibited a substantially lower rate of inosine formation in mature tRNA-Val (Fig. 4A and C). Thus, the V144M mutation appears to impair adenosine deaminase activity of endogenous ADAT2/3 complexes and uncovers a severe modification defect associated with the ADAT3-V144M variant in human individuals. Importantly, these findings suggest that individuals expressing only the ADAT3-V144M variant are likely to be compromised but not completely abolished in adenosine deaminase activity on wobble inosine-containing tRNA substrates *in vivo*.

### Purified ADAT2/3 complexes assembled with ADAT3-V144M exhibit defects in adenosine deaminase activity.

**FIG 5:**
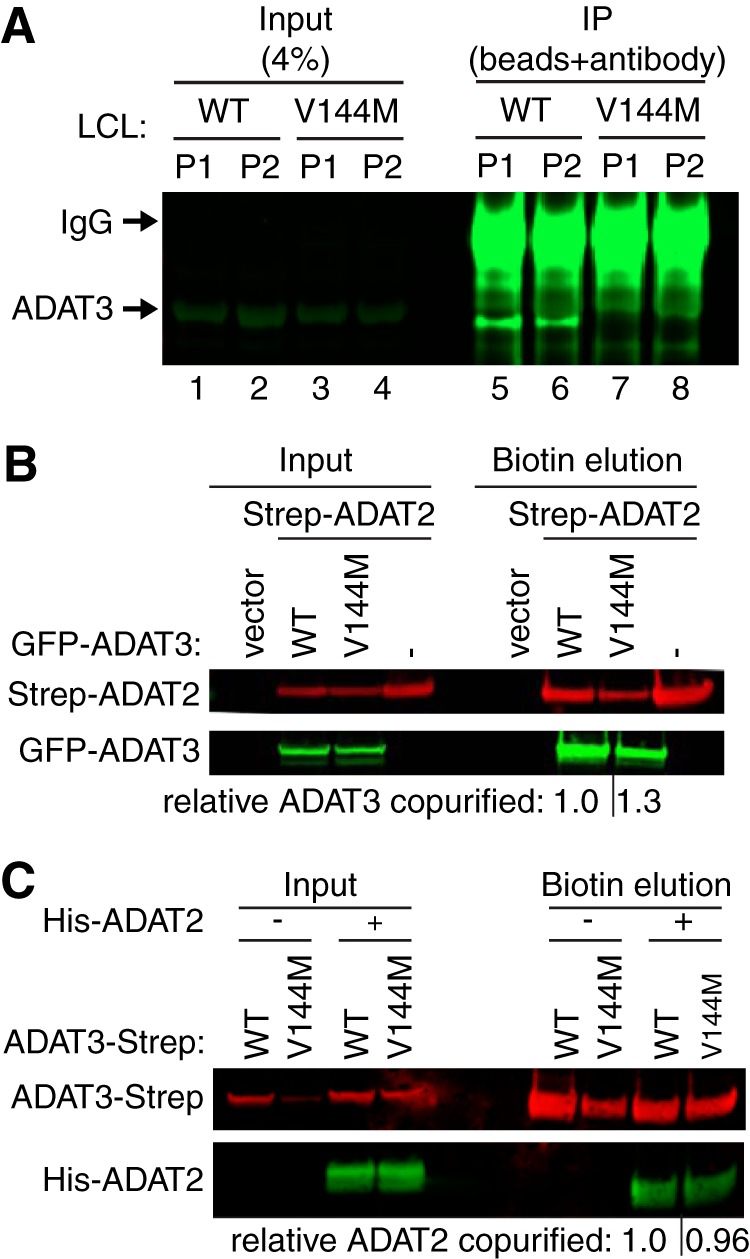
Purified ADAT2/3 complexes assembled with ADAT3-V144M exhibit defects in adenosine deaminase activity. (A) Immunoprecipitation (IP) of endogenous ADAT3. The input represents 4% of the starting extract used for IP. Arrows denote migration of ADAT3 and IgG. (B) ADAT3-V144M retains interaction with ADAT2. Shown are immunoblots for the indicated proteins from the input (5%) or biotin elutions of Strep-Tactin affinity purifications (10%) from HEK 293T cells transfected to express the Strep-tag alone (vector) or Strep-tagged ADAT2 with either GFP-ADAT3-WT or -V144M. “relative ADAT3 copurified” represents the ratio of the GFP-ADAT3 signal present in the eluted fraction normalized to the Strep-ADAT2 signal relative to ADAT3-WT. (C) ADAT3-WT and ADAT3-V144M copurify with similar levels of ADAT2. Shown are immunoblots for the indicated proteins from the input (5%) or biotin elutions from Strep-Tactin affinity purifications (20%) from HEK 293T cells transfected to express ADAT3-Strep-WT or ADAT3-Strep-V144M without or with His-ADAT2. “relative ADAT2 copurified” represents the ratio of the His-ADAT2 signal present in the eluted fraction normalized to the ADAT3-Strep signal relative to ADAT3-WT. Experiments for panels A through C were repeated three times, with comparable results.

Eukaryotic ADAT3 interacts with the catalytic subunit ADAT2 to form an active adenosine deaminase complex. We first attempted to characterize the interaction of ADAT2 with ADAT3 using immunoprecipitation (IP) with the same antibody used for detection of endogenous ADAT3. In both cellular extracts prepared from wild-type individuals, we were able to enrich for ADAT3 on antibody-coated beads (Fig. 5A, compare lanes 1 and 2 versus lanes 5 and 6). Unexpectedly, ADAT3 was undetectable in the IPs from extracts prepared from either ADAT3-V144M patient (Fig. 5A, lanes 7 and 8). The lack of IP for ADAT3-V144M was not due to decreased levels of starting material since similar levels of ADAT3 were present in the input extracts of either WT- or V144M-LCLs (Fig. 5A, lanes 1 to 4). The lack of ADAT3 recovery from V144M-LCL extracts was also observed using an independent preparation of cellular extract (J. Ramos and D. Fu, unpublished data). Since the polyclonal antibody was generated against full-length human ADAT3, the altered IP characteristics suggest that ADAT3-V144M adopts a different structure and/or interaction than ADAT3-WT, which reduces its antigenicity under native conditions. Another possibility is that the location of the V144M mutation represents the primary antigenic determinant for this polyclonal antibody.

Since the endogenous ADAT3-V144M variant was resistant to immunoprecipitation, we developed a purification system based upon the expression of tagged ADAT3 variants in HEK 293T human embryonic cells. To analyze the interaction between ADAT3-V144M and ADAT2, we coexpressed green fluorescent protein (GFP)-tagged ADAT3-WT or -V144M with ADAT2 tagged with the Twin-Strep-tag in HEK 293T human embryonic kidney cells. The Strep-tag allows for one-step affinity purification of Strep-tagged proteins on Strep-Tactin resin under native conditions followed by gentle elution with biotin to preserve any protein-protein interactions (40). After purification of Strep-ADAT2 on Strep-Tactin resin, we found that comparable levels of GFP-ADAT3-WT and GFP-ADAT3-V144M interacted with ADAT2 (Fig. 5B). These results indicate that ADAT3-V144M can still form a complex with ADAT2.

To validate the interaction between ADAT2 and ADAT3-V144M, we used a reciprocal approach in which we purified ADAT3 and examined the amount of copurifying ADAT2. For these assays, we transiently expressed either ADAT3-WT or -V144M fused to a carboxy-terminal Strep-tag for purification and elution of ADAT2/3. Since ADAT2 levels have been shown to be limiting for the formation of ADAT2/3 complexes in human cells (15), we coexpressed His-tagged ADAT2 with either ADAT3-Strep-WT or ADAT3-Strep-V144M to facilitate the detection of any associated ADAT2. After purification on Strep-Tactin resin, bound ADAT3 complexes were eluted with biotin and analyzed by immunoblotting. Using this approach, we detected the copurification of His-ADAT2 with either ADAT3-WT or ADAT3-V144M, consistent with the assembly of an ADAT2/3 complex from the expressed proteins (Fig. 5C). We detected comparable levels of His-ADAT2 that copurified with ADAT3-WT and -V144M, corroborating our finding that the ADAT3-V144M mutant maintains a similar interaction with ADAT2.

**FIG 6:**
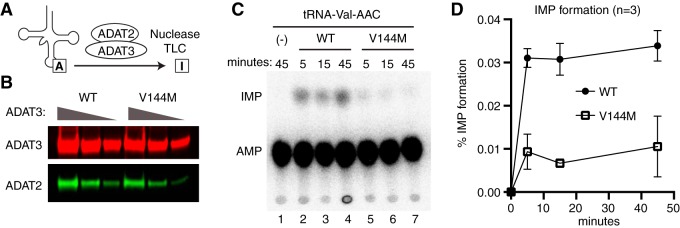
Purified ADAT2/3 complexes assembled with ADAT3-V144M exhibit defects in adenosine deaminase activity. (A) Schematic of the adenosine deaminase assay for inosine formation using *in vitro*-transcribed tRNA-Val-AAC. After incubation with ADAT2/3, labeled tRNA was digested with P1 nuclease, followed by separation of adenosine (A) from inosine (I) by thin-layer chromatography (TLC). (B) Immunoblot of purified ADAT2/3 complexes with 2-fold dilution series. The blot was probed for ADAT3-Strep and His-ADAT2. (C) Representative phosphorimager scan of TLC-separated nucleoside products from tRNAs incubated with buffer (−) or ADAT2/3 for the indicated times. The migration of IMP and AMP is indicated. (D) Quantification of IMP formation as a function of time for the indicated ADAT2/3 enzymes. Percent IMP formation represents the IMP/AMP plus IMP signal (*n* = 3).

We next employed the TLC-based IMP detection assay described above to investigate whether the V144M mutation affects adenosine deaminase activity of purified ADAT2/3 complexes on *in vitro*-transcribed tRNA (Fig. 6A). ADAT2/3 complexes were purified using a strategy identical to the one described above, using Strep-tagged versions of either ADAT3-WT or -V144M coexpressed in the presence of His-tagged ADAT2. The purified complexes were analyzed by immunoblotting for ADAT2 and ADAT3 to ensure that equivalent amounts of complexes were used for enzymatic assays (Fig. 6B). Using a time course to monitor inosine formation, we found that purified ADAT2/3 complexes assembled with ADAT3-WT exhibited robust adenosine deaminase activity on mature tRNA-Val-AAC, as evidenced by the formation of inosine (Fig. 6C lanes 2 to 4). Compared to ADAT2/3-WT complexes, purified ADAT2/3-V144M complexes were significantly diminished in adenosine deaminase activity on mature tRNA-Val-AAC (Fig. 6C and D). The activity defect detected with purified ADAT2/3 complexes assembled with ADAT3-V144M is consistent with the diminished adenosine deaminase activity of extracts prepared from V144M patient cells as described above. These findings reveal that while ADAT3-V144M can still associate with ADAT2 in human cells, the variant ADAT2/3-V144M complexes exhibit defects in adenosine deaminase activity.

### ADAT3-V144M exhibits an increased propensity to self-associate.

**FIG 7:**
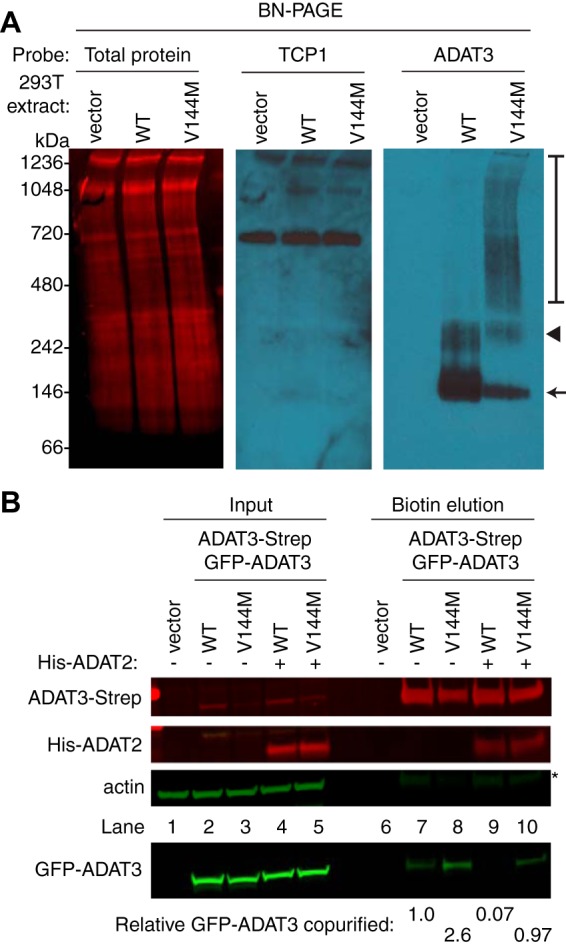
ADAT3-V144M exhibits an increased propensity to form higher-molecular-weight complexes indicative of aggregation. (A) Blue native polyacrylamide gel electrophoresis (BN-PAGE) analysis of ADAT3-GFP. Extracts prepared from HEK 293T cells expressing either ADAT3-WT or -V144M were separated on 3 to 12% BN-PAGE gels, followed by immunoblotting and probing with the indicated antibodies. The arrow and arrowhead point to predominant bands found in the WT and V144M extracts, while the bracket encompasses the high-molecular-weight signal detected in the ADAT3-V144M lane. Total protein indicates staining of the same blot to show loading of extracts. (B) Increased self-association of ADAT3-V144M. Shown are immunoblots for the indicated proteins from the input (5%) or biotin elutions from Strep-Tactin affinity purifications (20%) from HEK 293T cells transfected to express ADAT3-Strep-WT with GFP-ADAT3-WT or ADAT3-Strep-V144M with GFP-ADAT3-V144M in the absence or presence of ADAT2 coexpression. * represents the ADAT3-Strep signal from previous probing. The percentage of ADAT3 copurified represents the ratio of the GFP-ADAT3 signal present in the eluted fraction normalized to the Strep-ADAT3 signal relative to ADAT3-WT. Experiments for panels A and B were repeated three times.

Using the tagged-ADAT3 system, we next investigated the biochemical properties of ADAT3 complexes using blue native polyacrylamide gel electrophoresis (BN-PAGE), which has been used previously to characterize protein complexes (41, 42). After fractionation of human cell extracts by BN-PAGE and blot transfer, total protein staining revealed approximately equal loading and transfer of proteins from 66 to 1,236 kDa (Fig. 7A). To ensure that protein complexes were maintained during electrophoresis, we probed against the TCP1 subunit of the TRiC/CCT chaperonin complex, which is known to migrate as a high-molecular-weight complex on BN-PAGE gels (43). We detected the TRiC/CCT chaperonin complex migrating at ∼800 kDa, which is the expected molecular weight of the complex (Fig. 7A). Endogenous ADAT3 in HEK 293T cells was undetectable in human cell extracts by BN-PAGE immunoblotting, possibly due to masking of the epitope when ADAT3 is in complex with ADAT2 (Fig. 7A, vector). However, transiently expressed GFP-ADAT3-WT was detectable by BN-PAGE, with the majority of the signal concentrated in two bands migrating between the 100- and 250-kDa size markers (Fig. 7A, arrow and arrowhead). Based upon the molecular weight of ADAT3, the migration pattern of ADAT3-WT is consistent with ADAT3 homodimers or heterodimers with ADAT2. There is also the possibility that the upper band is an ADAT3 tetramer (Fig. 7A, arrowhead), which is a common property of cytidine and adenosine deaminases, including Escherichia coli TadA (44–46). ADAT3-V144M was also detectable by BN-PAGE as two bands migrating at molecular weights similar to those of the complexes detected in the ADAT3-WT sample although at lower levels than the WT (Fig. 7A, arrow and arrowhead). Notably, the ADAT3-V144M lane also contained high-molecular-weight complexes that exhibited an apparent molecular weight ranging from 400 to 1,000 kDa (Fig. 7A, bracket).

The high-molecular-weight complexes observed with ADAT3-V144M but not ADAT3-WT suggest that ADAT3-V144M has an increased propensity to self-associate or interact with additional proteins besides ADAT2. To test for self-oligomerization, we monitored the interaction of differentially tagged versions of wild-type ADAT3 with wild-type ADAT3 or of mutant ADAT3-V144M with mutant ADAT3-V144M. For these assays, we expressed either a WT version of GFP-ADAT3 with a WT form of ADAT3-Strep or a V144M mutant version of GFP-ADAT3 with the V144M mutant form of ADAT3-Strep (Fig. 7B, lanes 2 to 5) (40). We detected a low level of GFP-ADAT3-WT copurifying with ADAT3-Strep-WT, suggesting that ADAT3 could already be susceptible to aggregation, even in the wild-type state (Fig. 7B, lane 7). Notably, we found that purification of ADAT3-Strep-V144M led to an increase in the amount of copurifying GFP-ADAT3-V144M compared to ADAT3-WT with itself (Fig. 7B, compare lanes 7 and 8). Moreover, we found that coexpression of His-ADAT2 could suppress the self-oligomerization of ADAT3-WT with another ADAT3-WT while partially reducing the self-association of the ADAT3-V144M variant (Fig. 7B, lanes 9 and 10). We also note that purification of either ADAT3-WT or -V144M led to similar levels of copurifying ADAT2 (Fig. 7B, lanes 9 and 10), consistent with our findings described above showing that ADAT3-V144M maintains interactions with ADAT2. These results provide evidence that the V144M mutation causes a change in the ADAT3 conformation that increases the propensity of ADAT3 to misfold and self-associate if not properly assembled with ADAT2. Moreover, the ability of ADAT2 to prevent self-association of either ADAT3-WT or -V144M suggests a role for stoichiometric levels of ADAT2 and ADAT3 in promoting proper folding of ADAT3.

### ADAT3-V144M is targeted by the cytoplasmic HSP60 and TRiC chaperonin complexes.

**FIG 8:**
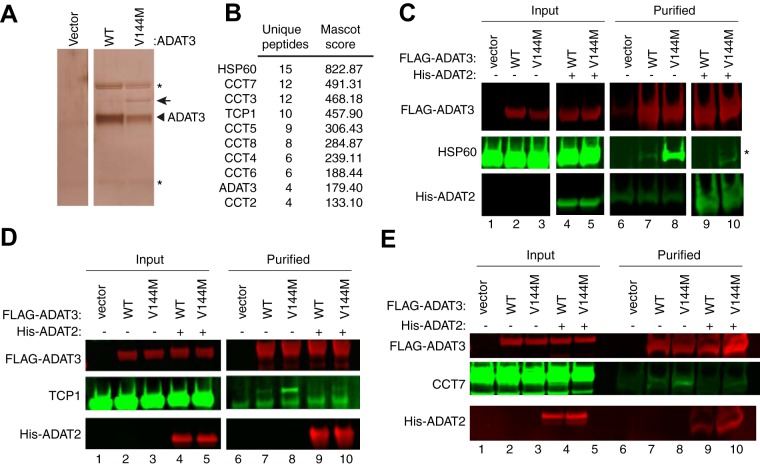
ADAT3-V144M is bound by the HSP60 and TRiC chaperonins. (A) Silver staining of eluted FLAG affinity purifications from HEK 293T cells expressing the FLAG tag alone (vector), FLAG-ADAT3-WT, or FLAG-ADAT3-V144M. The arrowhead represents FLAG-ADAT3, and the arrow represents a protein that specifically copurifies with ADAT3-V144M. (B) Chaperonin proteins identified by LC-MS proteomics specifically in ADAT3-V144M purifications. ADAT3 peptides are included for comparison. The number of unique peptides and Mascot score associated with each protein are noted. (C to E) Immunoblots for the indicated proteins from the input (5%) or FLAG affinity purifications (100%) from HEK 293T cells transfected to express FLAG-ADAT3-WT or -V144M without or with His-ADAT2. Purified samples represent SDS heat elutions of purified proteins retained on anti-FLAG antibody resin. IP immunoblots were repeated three times, with comparable results. * in panels A and C represents heavy and light chains of the anti-FLAG antibody used for affinity purification.

The altered biochemical properties of ADAT3-V144M suggest that the V144M mutation causes an altered protein conformation with an increased proclivity to self-associate or interact with additional proteins. We thus investigated whether ADAT3-V144M exhibited differential protein interactions compared to ADAT3-WT. For protein interaction analysis, we expressed either the WT or V144M versions of ADAT3 as fusion proteins with the FLAG epitope tag in HEK 293T human cells. While the Strep-tag was used as described above to allow for native elution of ADAT2/3 complexes using biotin, the FLAG tag was used for these studies since it allowed for more efficient purification of protein complexes, as we have shown previously (47, 48). Following immunoprecipitation, the purified samples were analyzed by SDS-PAGE and silver staining to identify ADAT3-interacting proteins. While no observable bands were found in a control purification of cells transfected with the vector alone, we could detect the purification of FLAG-ADAT3-WT or -V144M (Fig. 8A, arrowhead) along with an additional band at ∼60 kDa specifically enriched with the ADAT3-V144M purification (Fig. 8A, arrow). Analysis of the entire eluted samples from control and ADAT3 purifications by LC-MS validated the successful recovery of ADAT3-WT or ADAT3-V144M from cellular extracts (Fig. 8B; see also Table S1 in the supplemental material). Notably, LC-MS analysis also revealed the copurification of heat shock protein 60 (HSP60) and all eight subunits of the TCP1 ring complex (TRiC; also known CCT) with ADAT3-V144M but not ADAT3-WT (Fig. 8B and Table S1). HSP60 and TRiC subunits were identified among the top 20 best-scoring matches in the ADAT3-V144M purification. The HSP60 protein forms a homooligomeric chaperonin complex consisting of a double-heptameric ring that associates with misfolded proteins in the cytoplasm and mitochondria to provide an environment for protein refolding (37, 49–51). Similar to HSP60, TRiC is a major eukaryotic cytoplasmic chaperonin that is responsible for the correct folding of endogenous client proteins that are prone to misfolding (38, 50). The interaction of chaperonin complexes with ADAT3-V144M is consistent with a change in protein conformation and misfolding induced by the V144M mutation. We also note that peptides matching ADAT2 were not identified in the purifications of either ADAT3-WT or -V144M when purified without coexpression of ADAT2. The lack of copurification of ADAT2 with overexpressed ADAT3 agrees with previous findings that the levels of endogenous ADAT2 are limiting for the formation of an ADAT2/3 complex (12).

To verify and characterize the interaction between HSP60 and ADAT3-V144M, we performed co-IP experiments followed by immunoblotting. For a subset of these assays, we coexpressed His-tagged ADAT2 with either WT or V144M versions of FLAG-ADAT3 to investigate whether ADAT2 influences HSP60 interactions as described above. While a low level of HSP60 copurified with ADAT3-WT, we detected a significantly increased amount of HSP60 associated with ADAT3-V144M (Fig. 8C, lanes 7 and 8). Interestingly, the interaction between HSP60 and ADAT3-V144M could be greatly suppressed by coexpression with ADAT2 (Fig. 8C, lanes 9 and 10). The reduction in HSP60 association with ADAT3 by ADAT2 coexpression again demonstrates that assembly of ADAT2 with ADAT3 is likely to prevent misfolding and subsequent targeting by chaperonin complexes. Of note, similar levels of ADAT2 copurified with both ADAT3-WT and -V144M (Fig. 8C, lanes 9 and 10), further corroborating the results described above showing that the V144M mutation does not compromise the interaction between ADAT2 and ADAT3.

Using an analogous co-IP approach, we also found that the TRiC complex subunits TCP1 and CCT7 exhibited a significantly increased association with ADAT3-V144M compared to wild-type ADAT3 (Fig. 8D and E, compare lanes 7 and 8). Similar to the ADAT3 interaction with HSP60, we also found that coexpression of ADAT2 could suppress the association between the TRiC complex and ADAT3-WT while significantly reducing the amount of TRiC associated with ADAT3-V144M (Fig. 8D and E, compare lanes 7 and 8 to lanes 9 and 10). The targeting of either ADAT3-WT or -V144M by cellular chaperonin complexes suggests that ADAT3-WT is prone to misfolding, with the V144M mutation further exacerbating the misfolding phenotype. Furthermore, these studies provide additional evidence that ADAT2 facilitates the proper folding of ADAT3.

### ADAT3-V144M displays an aberrant subcellular localization that is suppressed by ADAT2 coexpression.

The studies described above suggest that the ADAT3-V144M mutation perturbs the folding and/or activity of the ADAT2/3 complex on particular tRNA substrates, thereby leading to reduced levels of wobble inosine modifications. Since wobble inosine modification has been proposed to occur in the nucleus and cytoplasm of eukaryotes (11, 12, 39), we next monitored whether the subcellular localization of endogenous ADAT3 was altered by the V144M mutation. We first tested patient LCLs via immunofluorescence microscopy. While we could detect a weak fluorescence signal within WT- and V144M-LCLs, the diffuse signal combined with the spherical morphology of LCLs precluded any definitive conclusion on the subcellular localization of either ADAT3-WT or -V144M (Ramos and Fu, unpublished).

**FIG 9:**
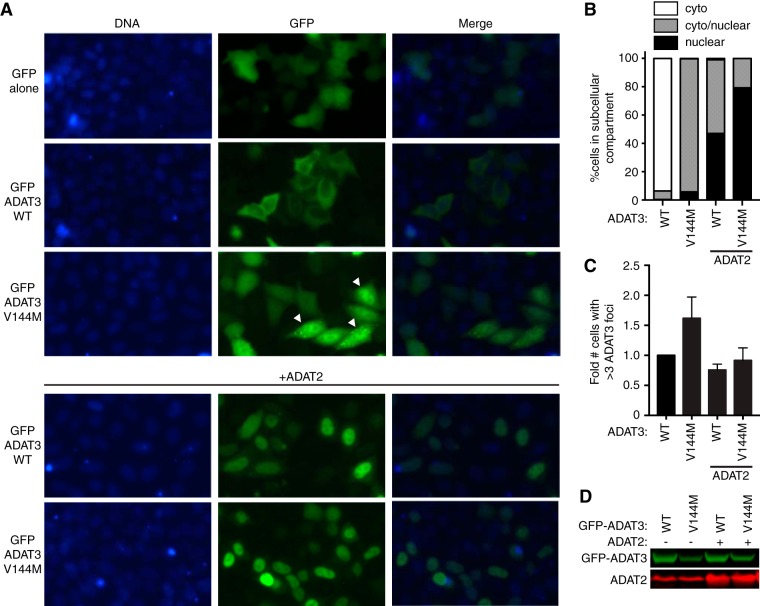
ADAT3-V144M displays an aberrant nucleocytoplasmic localization and increased susceptibility to form cytoplasmic aggregates. (A) Fluorescence microscopy images of GFP alone and GFP-tagged ADAT3-WT and -V144M expressed in HeLa cervical carcinoma cells. The bottom two rows exhibit cells cotransfected with untagged ADAT2. Nuclear DNA was stained with Hoechst stain, with merged images shown on the right. Arrowheads represent cells with >3 cytoplasmic foci of GFP-ADAT3. (B) Fraction of cells exhibiting GFP-ADAT3 that was either primarily cytoplasmic (cyto), similarly distributed between the cytoplasm and nucleus (cyto/nuclear), or primarily nuclear. (C) Fold change in the number of cells that exhibited more than three cytoplasmic foci of GFP-ADAT3. The fold change is expressed relative to ADAT3-WT without ADAT2 coexpression where 7% of cells exhibited more than three cytoplasmic foci. Experiments for panels B and C were repeated three times, with a minimum of 580 cells counted per experiment. (D) Immunoblot of GFP-ADAT3 expression without or with transient expression of ADAT2.

Due to the difficulty in visualizing ADAT3 in LCLs, the localization of ADAT3 was determined by microscopy of HeLa human cervical carcinoma cells transiently expressing ADAT3 fusion proteins with green fluorescent protein at the amino terminus (GFP-ADAT3). Whereas GFP alone displayed uniform accumulation in both the cytoplasm and nucleus of HeLa cells (Fig. 9A), the majority of cells expressing GFP-ADAT3-WT exhibited a diffuse cytoplasmic localization outlining the nucleus, with only a small percentage of transfected cells exhibiting a GFP-ADAT3 signal in the nucleus (Fig. 9A and B). The absence of nuclear localization for GFP-ADAT3-WT is likely due to the limiting amounts of the endogenous ADAT2 subunit that is required for the nuclear import of ADAT3 (12). In contrast, the ADAT3-V144M variant exhibited a distinct localization pattern with distribution in both the cytoplasm and nucleus rather than the primarily cytoplasmic localization of ADAT3-WT (Fig. 9A and B). In addition to aberrant nuclear localization, we detected an increased population of GFP-positive cells that exhibited discrete, cytoplasmic foci when transfected with the ADAT3-V144M variant (Fig. 9A and C). The accumulation of ADAT3-V144M into distinct cytoplasmic foci is consistent with the increased propensity of ADAT3-V144M to self-associate into large multimeric complexes as described above. Moreover, the steady-state levels of the GFP-ADAT3-V144M variant were lower than those of ADAT3-WT, suggesting that the aberrant subcellular localization pattern of ADAT3-V144M was not simply due to greater expression (Fig. 9D).

In contrast to the cytoplasmic localization of transiently expressed GFP-ADAT3-WT alone, the coexpression of ADAT2 with ADAT3-WT led to GFP-ADAT3-WT being localized to the nucleus, with only a minor proportion of the signal remaining in the cytoplasm (Fig. 9A and B), consistent with the observation that ADAT2 dimerization with ADAT3 is required for nuclear import of the ADAT2/3 complex (12). Similarly, we found that coexpression of ADAT2 with ADAT3-V144M could also induce the translocation of GFP-ADAT3-V144M into the nucleus (Fig. 9A and B). The ability of ADAT2 coexpression to induce the translocation of GFP-ADAT3-V144M into the nucleus indicates that ADAT3-V144M can still interact with ADAT2. However, while a slight, diffuse signal of GFP-ADAT3-WT remained in the cytoplasm even with ADAT2 coexpression, the ADAT3-V144M variant displayed much greater nuclear accumulation in the majority of cells. Remarkably, coexpression of ADAT2 with the ADAT3-V144M variant also reduced the percentage of cells with cytoplasmic GFP-ADAT3 foci to nearly wild-type levels (Fig. 9C).

**FIG 10:**
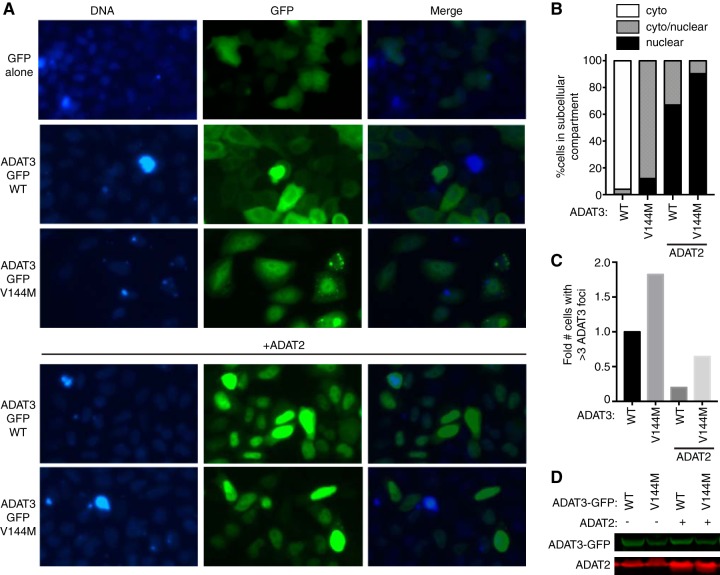
ADAT3-V144M displays an aberrant nucleocytoplasmic localization and increased susceptibility to form cytoplasmic aggregates also when tagged at the C terminus with GFP (ADAT3-GFP). (A) Fluorescence microscopy images of GFP alone and GFP-tagged ADAT3-WT and -V144M expressed in HeLa cervical carcinoma cells. Nuclear DNA was stained with Hoechst stain, with merged images shown on the right. (B) Fraction of cells exhibiting ADAT3-GFP that was either primarily cytoplasmic, similarly distributed between the cytoplasm and nucleus, or primarily nuclear. (C) Fold change in the number of cells that exhibited more than three cytoplasmic foci of GFP-ADAT3. For panels B and C, a minimum of 615 cells were counted per experiment. (D) Immunoblot of ADAT3-GFP expression without or with ADAT2 coexpression.

We also found that carboxy-terminally GFP-tagged ADAT3-V144M exhibited the same aberrant nucleocytoplasmic localization pattern and increased formation of cytoplasmic foci observed with N-terminal GFP-ADAT3-V144M (Fig. 10A to C). Similar to the results observed with N-terminally GFP-tagged ADAT3-V144M, ADAT2 coexpression could also suppress the increased levels of foci associated with carboxy-terminally GFP-tagged ADAT3-V144M (Fig. 10C and D). Altogether, these results uncover an aberrant subcellular localization pattern for ADAT3-V144M characterized by a perturbed nucleocytoplasmic distribution and increased formation of cytoplasmic foci that can be ameliorated by coexpression with ADAT2. The increased propensity to self-oligomerize, interaction with cytoplasmic chaperones, and formation of aberrant cytoplasmic foci exhibited by ADAT3-V144M provide evidence for a protein folding defect induced by the V144M mutation that reduces enzymatic activity and wobble inosine levels in the tRNAs of ID-affected human individuals.

## DISCUSSION

The molecular consequences of the ID-causing ADAT3-V144M mutation have previously been unknown. Here, we show that the ADAT3-V144M mutant accumulates to similar steady-state levels as ADAT3-WT and maintains an interaction with the ADAT2 subunit. However, the V144M mutation compromises the adenosine deaminase activity of ADAT2/3 complexes, increases the propensity of ADAT3 to oligomerize, and alters the subcellular localization properties of ADAT3. While the V144M mutation is a relatively minor change since valine and methionine represent amino acid residues with hydrophobic side chains, our studies uncover severe molecular defects associated with ADAT3-V144M that are likely to underlie the reduced wobble inosine levels detected in the tRNAs of ID-affected individuals with ADAT3-V144M mutations.

The changes in protein structure caused by the ADAT3-V144M mutation may alter the substrate recognition site of the ADAT2/3 complex, thereby affecting the binding or catalysis step of certain tRNA substrates. Intriguingly, studies with Trypanosoma brucei homologs of Tad2p/Tad3p have revealed a role for ADAT3 in substrate tRNA binding and coordination of a single zinc ion (36, 52). Moreover, we find that the ADAT3-V144M mutation has differential effects on wobble inosine levels, with certain tRNAs exhibiting a substantial decrease in tRNA modification and others displaying only a minor change. This differential effect could be due to the recognition mechanism of human ADAT2/3, which targets tRNA anticodon loops for inosine modification based upon their structural context rather than simply their sequence alone (53). Thus, ADAT2/3 complexes could have different specific activities on distinct tRNAs due to the additional structural features that affect protein-RNA binding and positioning in the active site. Further refinement using RNA binding assays and kinetics will provide insight into the specific effect of the V144M mutation on ADAT2/3 enzymatic activity that influences inosine modification levels *in vivo*.

The association of ADAT3-WT with cytoplasmic chaperonins suggests that endogenous ADAT3 could be prone to misfolding during translation or after release from the ribosome if not assembled with ADAT2. Consistent with our results, others have found that expression of soluble eukaryotic ADAT3 in E. coli requires coexpression of ADAT2 (10, 54). The increased tendency of ADAT3-V144M to form cytoplasmic foci suggests that the V144M mutation could further aggravate misfolding. Of note, structural studies have shown that methionine differs from other hydrophobic residues in that it can form noncovalent interactions with aromatic-containing residues such as tryptophan, tyrosine, or phenylalanine (55, 56). In addition, molecular modeling simulation experiments have identified that approximately one-third of solved protein structures contain a methionine-aromatic residue interaction (57). Thus, the replacement of a valine with methionine in the N-terminal extension of ADAT3 could lead to a nonspecific interaction with aromatic residues of another ADAT3 protein, leading to aggregation. Intriguingly, the perturbed nuclear localization and aggregation into discrete cytoplasmic foci exhibited by the ADAT3-V144M variant are reminiscent of other RNA binding proteins known to misfold and homooligomerize in neurological disorders, such as TDP-43 and TLS/FUS (58–62).

**FIG 11:**
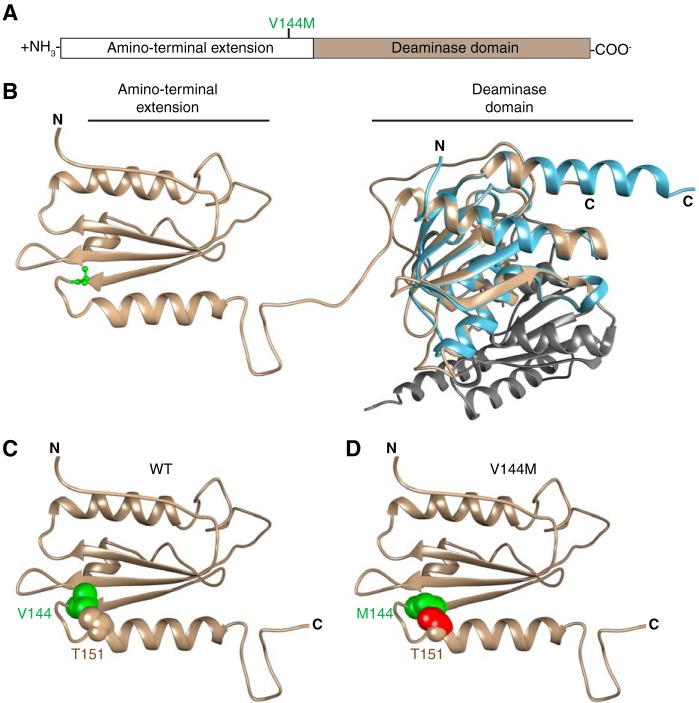
Predicted structure of ADAT3 and potential effects of the V144M mutation. (A) Schematic of ADAT3 with the location of the V144M mutation. (B) Based upon template-based tertiary structure prediction, ADAT3 is predicted to fold into two domains consisting of the amino-terminal extension and the deaminase domain. Valine 144 is highlighted in green in ball-and-stick form. The structure of the homodimeric E. coli TadA (PDB code 1Z3A) is aligned with the predicted C-terminal domain of ADAT3. Each monomer of TadA is shown in light blue and gray, respectively. (C) Amino-terminal extension of ADAT3, with the side chain of valine 144 shown in green in space-filling form. The side chain of threonine 151 is also shown in space-filling form in the same color as in the ribbon sequence. (D) Amino-terminal extension of ADAT3, with the V144M mutation shown in green. Atoms in the side chain of threonine 151 that clash with the methionine 144 side chain are shown in red.

To gain insight into the potential structural effects of the V144M mutation that could account for our results, we used *in silico* comparison of ADAT3 against the known structures of tRNA adenosine deaminases (46, 63–65). Based upon template-based tertiary structure prediction (66), ADAT3 is predicted to fold into two distinct domains coinciding with the N-terminal extension and the C-terminal deaminase motif, as previously predicted (2, 63) (Fig. 11A and B). Valine 144 lies within the N-terminal extension at the end of a three-stranded beta sheet immediately before a tight turn caused by the adjacent proline at position 145. Notably, we find that replacing the valine with methionine leads to a clash in van der Waals radii with a threonine residue (T151) of the neighboring helix after the turn (Fig. 11C and D). Thus, the valine-to-methionine mutation could alter the proper folding of ADAT3.

Due to the intricate dynamics of tRNA processing (67–69), alterations in ADAT3 nucleocytoplasmic localization by the V144M mutation provide another possible contributor to the decreased levels of wobble inosine tRNA modification in ID-affected individuals. The increased propensity of overexpressed ADAT3-V144M to localize to the nucleus suggests that there could be a relative decrease in the amount of cytoplasmic ADAT3 in ID-affected individuals with homozygous ADAT3-V144M mutations. The cytoplasmic population of ADAT3 assembled with ADAT2 could play a role in modifying tRNAs that have been exported without prior wobble inosine modification by nuclear ADAT2/3. Thus, the disruption of the nucleocytoplasmic ratio by the V144M mutation combined with the activity defect of ADAT2/3-V144M complexes could lead to the reduction in wobble inosine modification levels observed in the tRNAs of individuals homozygous for the ADAT3-V144M mutation. In addition, newly exported tRNAs lacking inosine could undergo retrograde transport back into the nucleus to be modified by nuclear ADAT2/3 (70, 71). Retrograde transport could play a critical role in providing at least enough tRNA wobble inosine modification for sufficient translation to sustain cell viability.

Based upon these findings, we hypothesize that a certain level of wobble inosine modification in particular tRNAs is necessary for the expression of cellular mRNAs that are critical for proper cellular physiology and human development. Consistent with this prediction, studies in the yeast Schizosaccharomyces pombe and the plant Arabidopsis thaliana have shown that a decrease in tRNA wobble inosine modifications leads to temperature sensitivity, cell cycle arrest, and growth retardation (9, 11). Moreover, genome-wide studies predict numerous highly expressed genes that are dependent upon ADAT2/3-catalyzed wobble inosine modification for translation (72). Thus, the V144M mutation could alter the cellular proteome in multiple tissues, with particularly acute effects in the brain on neural growth and differentiation.

## MATERIALS AND METHODS

### Human subjects.

Evaluation of affected members by a board-certified clinical geneticist included obtaining medical and family histories, clinical examination, neuroimaging, and clinical laboratory investigations. After obtaining written informed consent for enrollment in an institutional review board (IRB)-approved project (KFSHRC RAC number 2070023), venous blood was collected into EDTA and sodium heparin tubes for DNA extraction and establishment of lymphoblastoid cell lines (patients 11DG1699 and 09DG0640 and control subject 15DG0421), respectively. All studies abide by the principles of the Declaration of Helsinki.

### Plasmids.

The open reading frame for ADAT2 was PCR amplified from cDNA clone RC212395 (Origene) and cloned into pcDNA3.1 (Thermo Fisher) for expression as an untagged protein or as an N-terminal fusion protein with either the 6×His tag or Twin-Strep-tag (40). The open reading frame for human ADAT3 was PCR amplified from cDNA plasmid HsCD00326376 (PlasmID Repository, Harvard Medical School) and cloned into either pcDNA3.1-Strep-C, pcDNA3.1-3×FLAG-SBP, pcDNA3.1-N-EGFP, or pcDNA3.1-EGFP-C (73). The ADAT3-V144M variant was generated by Gibson mutagenesis and verified by Sanger sequencing.

### Cell culture.

HeLa S3 human cervical carcinoma and HEK 293T human embryonic kidney cell lines were cultured in Dulbecco’s minimal essential medium (DMEM) containing 10% fetal bovine serum (FBS), 2 mM l-alanyl-l-glutamine (GlutaMAX; Gibco), and 1% penicillin-streptomycin. Human lymphoblastoid cell lines were cultured in RPMI 1640 medium containing 15% fetal bovine serum, 2 mM l-alanyl-l-glutamine (GlutaMAX; Gibco), and 1% penicillin-streptomycin.

### Microscopy.

HeLa cells were plated at 2.5 × 10^5^ cells on a 6-well plate. Cells were transfected 1 day after plating with a total of 2.5 μg of DNA using Lipofectamine 3000. Cells were imaged at 48 h posttransfection on an Evos fluorescence microscopy imaging system (Thermo Fisher) for quantification of foci. For DNA staining, cells were washed twice with phosphate-buffered saline (PBS), incubated for 30 min at 37°C with PBS containing 10% FBS and 1 μM Hoechst stain, and then imaged. For quantification of cytoplasmic foci, 5 images of each well were taken, and the GFP-positive cells along with the cells containing more than 3 foci were counted in each of the 5 frames. The experiment was performed three times on N-terminally GFP-tagged ADAT3 with a minimum of 580 cells counted per experiment and independently verified by analysis in a blind manner. For C-terminally GFP-tagged ADAT3, the experiment was performed twice with independent verification. For quantification of nuclear ADAT3, data from each of the three experiments with N-terminally GFP-tagged ADAT3 were quantified using a minimum of 580 cells counted per experiment.

For visualization of ADAT3 in lymphoblastoid cells, 5 × 10^6^ cells were DNA stained with Hoechst stain for 30 min at 37°C. Cells were washed twice with PBS, fixed with 4% formaldehyde (15 min), permeabilized with 0.3% Triton X-100 (10 min), and blocked in 5% bovine serum albumin (BSA)–PBS, followed by probing with primary anti-ADAT3 antibody (catalog number H00113179-B01P; Abnova). Primary antibody was used at a dilution of 1:500 and left overnight, with shaking at 4°C. After washing with PBS containing 0.15% Tween 20, the secondary antibody (Alexa Fluor 633-conjugated goat anti-mouse IgG at 1:200; Invitrogen) was incubated for 1 h at 25°C. After washing with PBS with 0.15% Tween 20, coverslips were mounted onto glass microscope slides with Aqua-Poly/Mount (catalog number 18606-20; Polysciences, Inc.) and left to dry overnight. All cells for visualization of ADAT3 using the commercial mouse ADAT3 antibody were imaged on a Leica SP5 confocal microscope.

### Protein purification and analysis.

Transient transfection and cellular extract production were performed as previously described (47). In brief, 2.5 × 10^6^ HEK 293T cells were transiently transfected by calcium phosphate DNA precipitation with 10 to 20 μg of plasmid DNA, followed by preparation of the lysate by hypotonic freeze-thaw lysis at 48 h posttransfection. For anti-FLAG purification, the whole-cell extract from transiently transfected cell lines (1 mg of total protein) was rotated with 20 μl of anti-DYKDDDDK magnetic beads (TaKaRa BioUSA, Clontech, or Syd Labs) for 2 h at 4°C in lysis buffer (20 mM HEPES [pH 7.9], 2 mM MgCl_2_, 0.2 mM EGTA, 10% glycerol, 1 mM dithiothreitol [DTT], 0.1 mM phenylmethylsulfonyl fluoride [PMSF], 0.1% NP-40) with 200 mM NaCl. Resin was washed three times using the same buffer, followed by RNA extraction or protein analysis. Strep-tagged proteins were purified using MagSTREP “type 3” XT beads (5% suspension; IBA Lifesciences) under conditions similar to those for anti-FLAG purifications and eluted with desthiobiotin.

Protein identification was performed by the URMC Mass Spectrometry Resource Laboratory. Briefly, protein samples were reduced, alkylated, and digested in solution with trypsin, followed by purification and desalting on an analytical C_18_ column tip. Peptide samples were analyzed by high-performance liquid chromatography (HPLC) coupled with electrospray ionization on a Q Exactive Plus hybrid quadrupole-orbitrap mass spectrometer (Thermo Fisher). Protein identification through tandem mass spectral correlation was performed using SEQUEST and Mascot.

Cellular extracts and purified protein samples were fractionated on NuPAGE Bis-Tris polyacrylamide gels (Thermo Scientific), followed by transfer to an Immobilon FL polyvinylidene difluoride (PVDF) membrane (Millipore) for immunoblotting. For analysis of LCL extracts, 5 × 10^6^ lymphoblast cells were harvested, and proteins were extracted using radioisotope immunoprecipitation assay (RIPA) buffer (50 mM Tris HCl [pH 7.5], 1% NP-40, 0.5% sodium deoxycholate, 0.1% SDS, 150 mM NaCl, 2 mM EDTA). Antibodies against the following proteins were used: the FLAG epitope tag (catalog number A2220; Sigma), the 6×His tag (catalog number MA1-21315; Thermo Fisher), GFP (catalog number sc-9996; Santa Cruz Biotechnology), Strep-tag II (catalog number NC9261069; Thermo Fisher), ADAT3 (catalog number ab192987; Abcam), ADAT3 (catalog number H00113179-B01P; Abnova), ADAT2 (catalog number ab135429; Abcam), HSP60 (catalog number A302-845A; Bethyl Labs), CCT1 (catalog number sc-53454; Santa Cruz Biotechnologies), CCT7 (catalog number A304-730A-M; Bethyl Labs), and actin (catalog number MAB1501; EMD Millipore). Primary antibodies were detected using IRDye 800CW goat anti-mouse IgG (catalog number SA5-35521; Thermo Fisher), anti-rabbit IgG (catalog number SA5-35571; Thermo Fisher), or anti-rat IgG (catalog number 925-32219; Li-Cor Biosciences) or using IRDye 680RD goat anti-mouse IgG (catalog number 926-68070; Li-Cor Biosciences) or anti-rabbit IgG (catalog number 925-68071; Li-Cor Biosciences). Immunoblots were scanned using direct infrared fluorescence via the Odyssey system (Li-Cor Biosciences).

### Adenosine deaminase assays.

Internally radiolabeled tRNA substrates were prepared by T7 *in vitro* transcription of DNA templates generated by PCR amplification. Oligonucleotides containing the T7 promoter upstream of tRNA sequences were PCR amplified using Herculase II DNA polymerase or *Taq* DNA polymerase (New England Biolabs), followed by agarose gel purification of PCR amplification products. *In vitro* transcription was performed using Optizyme T7 RNA polymerase (Fisher Scientific) with 10 mM (each) UTP, CTP, and GTP; 1 mM ATP; and 250 μCi of [α-^32^P]ATP (800 Ci/mmol; 10 mCi/ml). *In vitro* transcription reaction mixtures were incubated at 37°C for 2 h, followed by DNase treatment and purification using RNA Clean and Concentrator columns (Zymo Research). Full-length tRNA transcripts were verified on a 15% polyacrylamide-urea gel stained with SYBR gold nucleic acid stain (Thermo Fisher). Before conducting enzymatic assays, all tRNA substrates were refolded by thermal denaturation at 95°C for 2 min in buffer containing final concentrations of 5 mM Tris (pH 7.5) and 0.16 mM EDTA, quick chilling on ice for 2 min, and refolding at 37°C in the presence of HEPES (pH 7.5), MgCl_2_, and NaCl.

For adenosine deaminase assays, ∼12 ng of the refolded tRNA substrate was incubated with either the lymphoblastoid extract or Strep-tag-purified ADAT3. Reaction mixtures were incubated at 37°C for 5, 15, and 45 min, and RNA was purified using RNA Clean and Concentrator columns. The tRNA was eluted in 10 μl of water and subjected to nuclease P1 digestion overnight in a total volume of 13 μl with 0.125 U of P1 in 250 mM ammonium acetate (pH 5.35). Half of the P1 nuclease-treated samples were spotted onto Polygram polyester cellulose MN 300 plates (Macherey-Nagel) run in solvent B (0.1 M sodium phosphate buffer [pH 6.8]–NH_4_ sulfate–*n*-propanol [100:60:2, vol/wt/vol]). Phosphorimaging was conducted on a Bio-Rad personal molecular imager, followed by analysis using NIH ImageJ software.

### RNA analysis.

RNA extraction was performed on 10 × 10^6^ human lymphoblastoid cells using TRIzol LS reagent (Thermo Fisher). For RT-PCR, total RNA (∼1.25 μg) was reverse transcribed for tRNA-Val-AAC using the Superscript IV enzyme followed by the QIAquick PCR purification kit. cDNA was then PCR amplified using Herculase II DNA polymerase (Agilent Genomics). The PCR product was gel extracted and analyzed by Sanger sequencing (ACGT, Inc.). The following primers were used: Val RT primer (TGTTTCCGCCTGGTTTTG), Val PCR primer F (GAACTAAGCTTGTTCAGAGTTCTACAGTCCGGACTACAAAGACCATGACGGTGATTATAAAGATCATGACATGTTTCCGTAGTGTAGTGGTTATCAC), and Val PCR primer R (CACTTGTTTCCGCCTGGTTTTGATCCAGGGACC).

For primer extension assays to monitor inosine modification status, oligonucleotides were 5′-end labeled and purified as previously described (74). In a 5-μl annealing reaction mixture, 0.25 to 1 pmol of labeled primers was annealed to 0.6 μg of bulk RNA by incubation for 3 min at 95°C, followed by slow cooling and incubation for 30 min at 50°C to 55°C. The annealing product was then extended using 64 U Superscript III (Invitrogen) in a 10-μl reaction mixture containing 1× first-strand buffer, 2 mM ddCTP, 0.5 mM each of the other deoxynucleoside triphosphates (dNTPs), and 10 mM MgCl_2_ at 50°C to 55°C for 1 h. Reactions were stopped by the addition of 2× RNA loading dye containing 98% formamide, 10 mM EDTA, 1 mg/ml bromophenol blue, and 1 mg/ml xylene cyanol and resolved on a 7 M urea–15% polyacrylamide gel, and the dried gel was imaged on a Typhoon phosphorimager and quantified as described previously (75). The primers for human tRNAs are as follows (lowercase letters refer to positions in the oligonucleotide probe that are not conserved among all tRNA sequences of a certain isoacceptor): Val(AAC)[50-36] (GGGaCCTTTCGCGTG), Ile(AAT)[50-36] (GCGaCCTTGGCGTTA), and Leu(AAG)[50-36] (GAAGAGACTGGAGCC).

### Phosphorimager analysis.

Phosphorimager scans were quantified using the Image Quant Fiji ImageJ processing package (76). For quantification of data from adenosine deaminase assays, the ratio of IMP/AMP pixel intensities was calculated using the peak signals from histogram plots of TLC images. Image quantification was performed using low-exposure scans that had peak signal intensities within the linear response range of the phosphorimager (data not shown).

For primer extension analysis of tRNA-Ile-AAU, peak signals from histogram plots of individual gel lanes were quantified based upon the area under the curve after adjustment to the background signal in an empty lane (data not shown). The percent signal intensity of each peak corresponding to an RT pause or stop was expressed relative to the total signal intensity of all quantifiable peaks between inosine and the guanosine stop.

### Blue native PAGE.

Protein extracts was prepared by resuspending transfected HEK 293T cells with lysis buffer (20 mM HEPES [pH 7.9], 2 mM MgCl_2_, 0.2 mM EGTA, 10% glycerol, 1 mM DTT, 0.1 mM PMSF) supplemented with 0.05% digitonin. Cells were incubated on ice for 5 min, followed by a 30-min centrifugation at 20,000 × *g* at 4°C. The supernatants representing soluble protein extracts were analyzed by blue native PAGE (BN-PAGE) according to the manufacturer’s protocol (Thermo Fisher). Briefly, samples were mixed with G-250 sample loading buffer and loaded onto 3 to 12% or 4 to 16% Novex Bis-Tris gels. Electrophoresis was performed at 150 V at 25°C until the dye front reached the bottom of the gel. Proteins were transferred and immunoblotted as described above, with the transfer time increased to 3 h.

### LC-MS analysis.

Total RNA was fractionated on an AdvanceBio column (300-Å pore size, 2.7-μm particle size, 7.8 by 300 mm; Agilent, Waldbronn, Germany) at 40°C using isocratic elution with 100 mM ammonium acetate at pH 7 as the mobile phase. The tRNA was collected in 1 ml 0.1 M NH_4_ acetate (NH_4_OAc) and vaporized by a SpeedVac until less than 50 μl remained in the vial. The tRNA fraction was ethanol precipitated, and tRNA pellets were resolved in 50 μl MilliQ water. Total tRNA (∼200 ng) was processed and analyzed by LC-MS as previously described (47, 77). The signal from each modified nucleoside was normalized to the levels of canonical nucleosides (1,000 nucleotides [nt]) for quantification. LC-MS measurements of modified nucleosides were carried out on three biological replicates, followed by statistical analysis based upon unpaired Student’s *t* test.

For absolute quantification of inosine, calibration solutions of inosine and the canonical nucleosides cytidine, uridine, guanosine, and adenosine were prepared with their respective synthetic standards (Sigma-Aldrich) and mixed to starting concentrations of 1 pmol/μl for the modified nucleoside and 100 pmol/μl for the canonical nucleosides. The resulting solution was diluted 1:1, and both dilutions were serially diluted 1:10 to a final concentration of 50 amol/μl for the modified nucleoside and 5 fmol/μl for the canonical nucleosides. A 1/10 volume of 10× yeast stable isotope-labeled internal standards (SILIS) was added to each calibration solution. Ten microliters of each calibration solution was subjected to LC-MS analysis before sample analysis using the same method as the one used for the samples.

The limit of detection (defined as a SILIS/nucleoside (S/N) ratio of >3) and limit of quantification (defined as an S/N ratio of >10) of inosine were 5 fmol. The MS peak areas of the modified nucleoside and canonicals were divided by the MS peak area of the corresponding SILIS and plotted over the amount of injected material. The slope of the linear regression resulted in the relative response factors and was used to determine the amount of inosine in the analyzed samples. In a last step, the amounts of modified nucleoside (in picomoles) were divided by the sum of canonicals to receive the modification percentage per 1,000 canonical nucleosides. Mass spectrometry parameters are shown in Table 1.

**TABLE 1:** Mass spectrometry parameters of all analyzed nucleosides

Compound^*a*^	Precursor ion (*m/z*)	Product ion (*m/z*)	Ret^*b*^ time (min)	Δ ret time (min)	Fragmentor voltage (V)	Collision energy (eV)	Cell accelerator voltage (V)	Polarity
A	268	136	5.1	1	110	21	5	Positive
A SILIS	278	141	5.1	1	110	21	5	Positive
C	244	112	2	1	175	13	5	Positive
C SILIS	253	116	2	1	175	13	5	Positive
Cm	258	112	3.7	1	180	9	5	Positive
Cm SILIS	270	116	3.7	1	180	9	5	Positive
G	284	152	4	1	95	17	5	Positive
G SILIS	294	157	4	1	95	17	5	Positive
Gm	298	152	4.8	1	100	9	5	Positive
Gm SILIS	311	157	4.8	1	100	9	5	Positive
I	269	137	3.8	1	100	9	5	Positive
I SILIS	279	142	3.8	1	100	9	5	Positive
m1A	282	150	3.5	1	110	21	5	Positive
m1A SILIS	295	158	3.5	1	110	21	5	Positive
m1G	298	166	4.7	1	105	13	5	Positive
m1G SILIS	311	174	4.7	1	105	13	5	Positive
m22G	312	180	5.5	1	105	13	5	Positive
m22G SILIS	328	191	5.5	1	105	13	5	Positive
m2G	298	166	4.9	1	95	17	5	Positive
m2G SILIS	311	174	4.9	1	95	17	5	Positive
m5C	258	126	3.5	1	185	13	5	Positive
m5C SILIS	270	133	3.5	1	185	13	5	Positive
m5U	259	127	4	1	95	9	5	Positive
m5U SILIS	271	134	4	1	95	9	5	Positive
m6A	282	150	6.4	1	125	17	5	Positive
m6A SILIS	295	158	6.4	1	125	17	5	Positive
m7G	299	167	3.6	1	105	14	5	Positive
m7G SILIS	311	174	3.6	1	105	14	5	Positive
U	245	113	2.7	1	95	5	5	Positive
U SILIS	254	117	2.7	1	95	5	5	Positive
Y	245	209	1.6	1	90	5	5	Positive
Y SILIS	254	218	1.6	1	90	5	5	Positive

a SILIS, stable isotope-labeled internal standards; Cm, 2′-*O*-methylcytidine; Gm, 2′-*O*-methylguanosine; I, inosine; m1A, 1-methyladenosine; m1G, 1-methylguanosine; m22G, *N*^2^,*N*^2^-dimethylguanosine; m5C, 5-methylcytosine; m5U, 5-methyluridine; m6A, *N*^6^-methyladenosine; m7G, *N*^7^-methyladenosine.

b Ret, retention.

## Supplementary Material

Supplemental file 1
